# Inter-stage right ventricular remodeling in hypoplastic left heart syndrome

**DOI:** 10.1186/1532-429X-13-S1-P205

**Published:** 2011-02-02

**Authors:** Aaron Bell, Hannah R Bellsham-Revell, Philipp Beerbaum, Reza Razavi, Gerald Greil

**Affiliations:** 1 Guy's and St Thomas' Foundation NHS Trust, London, UK; 2 King's College London, London, UK

## Background

Magnetic resonance imaging (MRI) can be used as a means of pre-operative evaluation of the cardiac anatomy and function in patients with hypoplastic left heart syndrome (HLHS) prior to superior and total cavo-pulmonary connection (TCPC). Serial MRI evaluation prior to each cavo-pulmonary palliation allows us to demonstrate the remodelling of the right ventricle.

## Methods

A retrospective analysis of the serial MRI scans of all patients with HLHS who had undergone palliation through to completion of Fontan was undertaken. Re-analysis was undertaken by two members of the research team who had previously shown good intrauser variability. Ethical and institutional approval was obtained. All scans had been performed with general anaesthesia on a Philips 1.5 Tesla Intera MRI scanner.

## Results

51 patients had undergone completion of Fontan and had MRI scans performed prior to each cavo-pulmonary connection available for analysis. Median (range) age in years at the first MRI, superior cavopulmonary connection, pre-TCPC MRI and TCPC was 0.44 (0.12-1.02), 0.57 (0.13-1.37), 2.80 (1.80-5.52), 3.26 (2.28-6.76) respectively. Right ventricular (RV) volumes indexed to body surface area are demonstrated in table 1. There was a marked reduction in indexed RV end diastolic volume (iEDV), indexed RV end systolic volume (iESV). Indexed stroke volume (iSV) remained constant, but indexed cardiac output (iCO) fell, likely due to the decrease in heart rate (HR) between the two scans. There was an increase in global ejection fraction (EF).

**Table 1:** 

	Heart Rate median (range)	iEDV (ml/m2) median (range)	iESV median (range)	iSV median (range)	iCO median (range)	EF median (range)
MRI prior to superior cavo-pulmonary connection	115 (83-150)	91.90 (48.67-213.63)	45.13 (15.31-126.5)	47.33 (19.91-105.77)	5.12 (2.49-13.36)	51.4 (28.4-72.4)
MRI prior to total cavo-pulmonary connection	85 (57-133)	84.45 (46.42-152.10)	36.12 (14.38-64.97)	48.02 (32.02-98.73)	4.15 (3.01-8.20)	59 (43.2-76.2)

## Conclusions

The right ventricle undergoes significant remodelling between palliative stages. Reduction in the volume loading by replacing a shunt dependent circulation with a cavo-pulmonary connection significantly alters right ventricular volumes and improves function.

